# Linking Local-Level Chronic Disease and Social Vulnerability Measures to Inform Planning Efforts: A COPD Example

**DOI:** 10.5888/pcd20.230025

**Published:** 2023-08-31

**Authors:** Susan A. Carlson, Kathleen B. Watson, Sarah Rockhill, Yan Wang, Magdalena M. Pankowska, Kurt J. Greenlund

**Affiliations:** 1 Division of Population Health, National Center for Chronic Disease Prevention and Health Promotion, Centers for Disease Control and Prevention, Atlanta, Georgia; 2 Geospatial Research, Analysis, and Services Program, Office of Innovation and Analytics, Centers for Disease Control and Prevention, Atlanta, Georgia; 3 Oak Ridge Institute for Science and Education, Research Participation Program, Division of Population Health, National Center for Chronic Disease Prevention and Health Promotion, Centers for Disease Control and Prevention, Atlanta, Georgia

## Abstract

**Introduction:**

Data are publicly available to identify geographic differences in health outcomes, including chronic obstructive pulmonary disease (COPD), and social vulnerability; however, examples of combining data across sources to understand disease burden in the context of community vulnerability are lacking.

**Methods:**

We merged county and census tract model-based estimates of COPD prevalence from PLACES (www.cdc.gov/PLACES) with social vulnerability measures from the Centers for Disease Control and Prevention/Agency for Toxic Substances and Disease Registry Social Vulnerability Index (https://www.atsdr.cdc.gov/placeandhealth/svi), including 4 themes (socioeconomic, household composition and disability, minority status and language, and housing type and transportation), and the overall Social Vulnerability Index (SVI). We used the merged data set to create vulnerability profiles by COPD prevalence, explore joint geographic patterns, and calculate COPD population estimates by vulnerability levels.

**Results:**

Counties and census tracts with high COPD prevalence (quartile 4) had high median vulnerability rankings (range: 0–1) for 2 themes: socioeconomic (county, 0.81; tract, 0.77) and household composition and disability (county, 0.75; tract, 0.81). Concordant high COPD prevalence and vulnerability for these themes were clustered along the Ohio and lower Mississippi rivers. The estimated number of adults with COPD residing in counties with high vulnerability was 2.5 million (tract: 4.7 million) for the socioeconomic theme and 2.3 million (tract: 5.0 million) for the household composition and disability theme (high overall SVI: county, 4.5 million; tract, 4.7 million).

**Conclusion:**

Data from 2 publicly available tools can be combined, analyzed, and visualized to jointly examine local COPD estimates and social vulnerability. These analyses can be replicated with other measures to expand the use of these cross-cutting tools for public health planning.

**Summary**

**What is already known on this topic?**

Data are available to identify geographic differences in health outcomes and community-wide measures of social determinants of health. Examples of how to combine and use data across different sources are lacking.

**What is added by this report?**

We demonstrated how prevalence estimates of chronic obstructive pulmonary disease from PLACES and social vulnerability measures from the Centers for Disease Control and Prevention/Agency for Toxic Substances and Disease Registry Social Vulnerability Index can be jointly examined. We provide examples of tables, figures, and maps that can be created from the combined data.

**What are the implications for public health practice?**

These analyses can be replicated to examine different health measures or for more focused geographies to inform public health planning and expand the use of these cross-cutting tools.

## Introduction

Social determinants of health (SDOH) are conditions in the environments where people are born, live, learn, work, and play that affect a wide range of health, function, and quality-of-life outcomes (1). Incorporating area-level SDOH measures into electronic records has been promoted as a strategy to advance patient and population health (2,3). For public health planning, tools are available to identify geographic differences in health outcomes, including chronic obstructive pulmonary disease (COPD), and community-wide measures of intermediary determinants of SDOH, including social vulnerability measures (4–10). 

Social vulnerability measures are factors related to community-level income, education, race or ethnicity, household composition, disability, housing, and transportation, as well as composite social vulnerability indexes (SVIs) (6). SVIs were created to help public health professionals and local planners better prepare for and respond to emergency events like hurricanes, disease outbreaks, or exposure to dangerous chemicals (11). The benefit of SVIs in planning for nonemergent events has been recently highlighted (12); however, examples of how to combine and examine health and social vulnerability data across different sources to use for public health planning are lacking.

Jointly examining local health and social vulnerability data can improve understanding of the interplay of these issues in the community setting and can enhance community planning strategies addressing chronic conditions, such as COPD. For example, local, state, and federal planning agencies and partner organizations can use these data to determine distribution of COPD-related resources and programming that address issues related to the cost of treatment in communities where socioeconomic vulnerabilities are highest. By combining these 2 types of data, it may also be possible to identify local areas where high levels of COPD prevalence occur with high social vulnerability. Identifying these areas can help inform allocation of appropriate resources and promote engagement of cross-sector partners like education, transportation, and housing, with the shared goal of improving people's environments and their health.

Our study demonstrates how COPD prevalence estimates from PLACES (www.cdc.gov/PLACES) and social vulnerability measures from the Centers for Disease Control and Prevention/Agency for Toxic Substances and Disease Registry Social Vulnerability Index (CDC/ATSDR SVI) (https://www.atsdr.cdc.gov/placeandhealth/svi) can be jointly examined. We first observed the correlations between COPD prevalence and CDC/ATSDR SVI variables at 2 geographic levels, county and census tract. Two geographic levels were examined because practitioners may be interested in how results vary depending on geographic level. Next, we showed how to 1) use these data to create a social vulnerability profile for areas with low, moderate, and high COPD prevalence; 2) jointly explore geographic patterns in these estimates by using bivariate maps; and 3) generate estimates for the prevalence and number of adults with COPD living in counties and tracts with low, moderate, and high vulnerability. We illustrate how publicly available data can be used to implement these types of analyses, and we provide tabular and graphic summaries that can inform public health planning discussions. We present findings at the national level, but analyses can be limited to a more focused geography (eg, counties within a specific state). We conclude with a discussion of key issues to consider when jointly examining these data.

## Methods

### PLACES: Local Data for Better Health

PLACES (www.cdc.gov/PLACES) provides annual model-based estimates for counties, incorporated and census-designated places, census tracts, and ZIP Code Tabulation Areas (ZCTAs) across the US for 29 chronic disease–related measures, including a measure of COPD prevalence (5). PLACES estimates are generated by applying a multilevel model and poststratification approach to Behavioral Risk Factor Surveillance System (BRFSS) data. BRFSS is a telephone survey designed to provide state-level estimates and includes more than 400,000 respondents each year from 50 states, the District of Columbia, and participating US territories (13); it is not designed to provide direct estimates of prevalence below the state level.

We downloaded county and census tract estimates of COPD prevalence from the 2020 release from the PLACES data portal. Estimates were generated by a multilevel model with COPD as the dependent variable that included individual-level fixed effects for age, sex, race or ethnicity, and education from 2018 BRFSS data; an area-level fixed effect for county-level percentage of the adult population below 150% of the federal poverty level from the American Community Survey (ACS), 2014**–**2018; and state- and county-level random effects. The model was applied to 2018 census county population estimates for county estimation and 2010 census decennial tract population counts for tract estimation. More detailed information on the PLACES methodology is available on the website and elsewhere (5,14). COPD prevalence estimates were available for all 3,142 counties and 72,337 of the 73,057 census tracts (PLACES estimates are available for tracts with a 2010 population count ≥50). When examined categorically, COPD prevalence estimates were grouped on the basis of quartiles: low (quartile 1: county, 3.5%–7.3%; tract, 1.1%–5.3%), moderate (quartiles 2 and 3: county, 7.4%–10.6%; tract, 5.4%–8.9%), and high (quartile 4: county, 10.7%–19.7%; tract, 9.0%–26.7%).

### Centers for Disease Control and Prevention/Agency for Toxic Substances and Disease Registry Social Vulnerability Index (CDC/ATSDR SVI)

The CDC/ATSDR SVI (https://www.atsdr.cdc.gov/placeandhealth/svi) uses ACS data to determine the social vulnerability of US counties and census tracts. ACS is a nationwide survey conducted by the US Census Bureau to produce sample-based estimates across multiple geographies (15). The 2018 CDC/ATSDR SVI uses ACS data related to 15 individual components grouped into 4 themes: socioeconomic (below poverty, unemployed, income, no high school diploma), household composition and disability (aged ≥65 y, aged ≤17 y, civilian with a disability, single-parent household), minority status and language (minority person, aged ≥5 y and speaks English “less than well”), and housing type and transportation (multi-unit structures, mobile homes, crowding, no vehicle, group quarters) (Table 1) (6,11). 

**Table 1:** Correlation Between PLACES COPD Prevalence and CDC/ATSDR SVI Components, Themes, and Overall SVI at the County and Tract Level

Name of component, theme, or overall index^a^	Description	County (n = 3,142)^b^		Census tract (n = 72,337)^c^	
		Median estimate^d^	Spearman correlation with COPD prevalence^e^	Median estimate^d^	Spearman correlation with COPD prevalence^e^
**Socioeconomic theme**	Combined rank of components^f^	NA	0.69	NA	0.61
Below poverty	Population below poverty line, %	14.7	0.61	12.0	0.55
Unemployed	Unemployment rate, %	5.4	0.46	5.3	0.36
Income	Per capita annual income, $	26,245	0.67	28,555	0.59
No high school diploma	People aged ≥25 y with no high school diploma, %	12.1	0.60	10.1	0.54
**Household composition and disability theme**	Combined rank of components^f^	NA	0.60	NA	0.70
Aged ≥65 y	People aged ≥65 y, %	18.0	0.36	15.2	0.32
Aged ≤17 y	People aged ≤17 y, %	22.3	−0.17	22.2	0.05
Civilian with a disability	Noninstitutionalized civilian population with a disability, %	15.4	0.75	12.5	0.74
Single-parent households	Single parent households with children aged <18 y, %	8.1	0.14	7.8	0.31
**Minority status and language theme**	Combined rank of components^f^	NA	−0.28	NA	−0.19
Minority	Minority population (excluding non-Hispanic White), %	16.1	−0.15	29.4	−0.12
Aged ≥5 y who speaks English “less than well”	People aged ≥5 y who speak English “less than well,” %	0.7	−0.36	1.3	−0.21
**Housing type and transportation theme**	Combined rank of components^f^	NA	0.13	NA	0.25
Multi-unit structures	Multi-unit structure with 10 or more units, %	2.9	−0.55	4.5	−0.22
Mobile homes	Mobile home, %	10.9	0.61	0.7	0.45
Crowding	Housing units with more people than rooms, %	1.9	0.02	1.8	0.04
No vehicle	Households with no vehicle available, %	5.7	0.34	5.3	0.29
Group quarters	People living in group quarters, %	2.0	−0.05	0.2	0.08
**Overall SVI**	Combined rank of themes^f^	NA	0.48	NA	0.51

Abbreviations: ATSDR, Agency for Toxic Substances and Disease Registry; CDC, Centers for Disease Control and Prevention; COPD, chronic obstructive pulmonary disease; NA, not applicable; SVI, social vulnerability index.

a The 2018 CDC/ATSDR SVI includes data for 15 individual components grouped into 4 themes (socioeconomic, household composition and disability, minority status and language, and housing type and transportation) which are then summarized with an overall SVI.

b A total of 3,141 counties for 3 components (below poverty, unemployed, and income), the socioeconomic theme, and the overall SVI because of missing data.

c The tract-level count ranged from 72,140 to 72,323 (depending on the variable) because of missing data.

d Median values are based on raw estimates of the individual components. Median values are not applicable to the 4 themes or the overall SVI because these measures are composites of multiple components.

e Correlations of ≥0.035 for counties and ≥0.0075 for tracts are significant.

f For each of the 4 themes, percentiles for the components each theme comprises were summed and then used to determine theme-specific percentile rankings. For the overall SVI, the sums for each theme were totaled, counties or tracts were ordered for the overall sum, and then the overall percentile rankings were calculated. Spearman correlations of the ranked value with COPD prevalence are presented.

Raw estimates and percentile rankings from the 2018 CDC/ATSDR SVI (released in 2020) for each county and tract were downloaded from the website. For the individual components, raw estimates were based on the ACS 2014**–**2018, and rankings were created based on percentiles of raw estimates (range 0–1 [higher values indicating greater vulnerability]). Percentiles for the components of each theme were summed and the totals were used to determine theme-specific percentile rankings. For overall SVI, the sums for each theme were totaled, and this sum was used to create rankings and determine overall percentile rankings. Maps at the county and tract level are provided for these rankings via the CDC/ATSDR SVI Interactive Map application (6). When examined categorically, rankings were grouped on the basis of either the county or tract: low vulnerability (quartile 1 [0 to ≤0.25]), moderate vulnerability (quartiles 2 and 3 [>0.25 to ≤0.75]), and high vulnerability (quartile 4 [>0.75 to 1.00]).

### Statistical analysis

Analyses were conducted separately for county and tract data. Spearman correlations were calculated to examine the strength of the relationships between continuous COPD prevalence and the ranking percentile value of each individual component, 4 themes, and overall SVI. Correlations were statistically tested by using a 2-tailed *t* statistic to determine whether they were significantly different from zero; correlations were described as strong (>0.50), moderate (0.30**–**0.50), or weak (<0.30) (16). Median raw estimates for individual components and median percentile ranking values for the components, 4 themes, and overall SVI were calculated, stratified by COPD prevalence categories. We estimated median raw COPD prevalence, population weighted prevalence, and population estimates of the number of adults living with COPD, stratified by 3 categories of vulnerability, for the themes and the overall SVI. Population weighted prevalence was estimated from the number of adults with COPD divided by the total number of adults (multiplied by 100%) for each level of vulnerability. By using a median test for 2 independent samples across 3 pairs (low vs moderate, low vs high, and moderate vs high), we performed pairwise comparisons of median values of all components, themes, and overall SVI between COPD prevalence categories; we also made pairwise comparisons of median values of COPD prevalence between 3 vulnerability categories for the themes and overall SVI. We jointly mapped COPD prevalence and vulnerability levels by using 2 approaches: 1) cross classified into 9 categories representing a combination of COPD prevalence with each theme and overall SVI (each categorized into 3 levels (17); 2) cross classified into 2 categories of concordance (ie, high prevalence and high vulnerability, low prevalence and low vulnerability); 2 categories of discordance (ie, high prevalence and low vulnerability, low prevalence and high vulnerability); and all other areas were categorized in a fifth category. We used SAS version 9.4 (SAS Institute) to conduct all analyses.

## Results

Correlations between COPD prevalence and individual components, themes, and the overall SVI were in similar ranges for county and tract estimates (Table 1). The relationship between COPD prevalence and the overall SVI index was moderate (Spearman *r* = 0.48 [county], 0.51 [tract]). Correlations were strong for the socioeconomic theme, and strong to moderate for all components within the theme. Correlations were strong for the household composition and disability theme although correlations differed across components. Correlations were negative and moderate at the county level and weak at the tract level for the minority status and language theme with correlations moderate to weak for both components within the theme. Correlations were weak for the housing type and transportation theme with variability across components.

Median values for the components, themes, and the overall SVI were examined by COPD prevalence category (Table 2). Median values increased as COPD prevalence increased for the socioeconomic and household composition and disability themes and the overall SVI. Counties and tracts with high COPD prevalence can be categorized as having high median vulnerability percentile rankings for the socioeconomic and household composition and disability themes. For the minority status and language theme, values decreased as COPD prevalence increased. Several components showed a pronounced difference (percentile rank absolute difference >0.50) between counties and tracts with high versus low COPD prevalence. Counties and tracts with high COPD prevalence had a pronounced higher percentage below poverty, no high school diploma, civilian with a disability, and housing units that are mobile homes; these counties and tracts also had a pronounced lower per capita income and a lower percentage of multi-unit structures (difference was not pronounced for tracts).

**Table 2:** Median Percentile Rank and Raw Estimate of Components, Themes, and Overall SVI by County and Census Tract — Low, Moderate, and High COPD Prevalence Levels

Component, theme, or overall index^a^	Median values by COPD prevalence category^b^					
	County (n = 3,142)^c^			Census tract (n = 72,337)^d^		
	Low (3.5–7.3)	Moderate (7.4–10.6)	High (10.7–19.7)	Low (1.1–5.3)	Moderate (5.4–8.9)	High (9.0–26.7)
**Socioeconomic theme**	0.19	0.47	0.81	0.21	0.47	0.77
Below poverty						
Rank, percentile	0.22	0.46	0.79	0.24	0.47	0.76
Raw, %	10.5	14.2	20.0	6.4	11.2	21.2
Unemployed						
Rank, percentile	0.29	0.47	0.73	0.34	0.47	0.72
Raw, %	4.3	5.3	7.0	4.1	5.1	7.6
Income						
Rank, percentile	0.19	0.48	0.80	0.19	0.47	0.76
Raw, $	31,648	26,583	22,023	41,699	29,346	21,528
No high school diploma						
Rank, percentile	0.20	0.47	0.78	0.20	0.47	0.73
Raw, %	8.1	11.7	18.1	4.6	9.6	16.8
**Household composition and disability theme**	0.19	0.51	0.75	0.19	0.51	0.81
Aged ≥65 y						
Rank, percentile	0.22	0.54	0.60	0.31	0.52	0.63
Raw, %	15.1	18.5	19.1	12.1	15.6	17.4
Aged ≤17 y						
Rank, percentile	0.60	0.49	0.43	0.49	0.49	0.52
Raw, %	23.1	22.3	21.9	22.2	22.1	22.5
Civilian with a disability						
Rank, percentile	0.16	0.51	0.81	0.18	0.49	0.83
Raw, %	11.6	15.6	19.7	8.2	12.5	18.5
Single-parent households						
Rank, percentile	0.41	0.51	0.54	0.32	0.51	0.64
Raw, %	7.7	8.2	8.4	5.6	8.1	10.1
**Minority status and language theme**	0.63	0.51	0.37	0.58	0.50	0.41
Minority						
Rank, percentile	0.56	0.50	0.39	0.56	0.48	0.45
Raw, %	19.7	16.3	11.6	35.3	27.7	25.6
Aged ≥5 y and speaks English less than well						
Rank, percentile	0.66	0.46	0.26	0.57	0.52	0.34
Raw, %	1.3	0.7	0.4	1.9	1.5	0.6
**Housing type and transportation theme**	0.45	0.47	0.56	0.36	0.50	0.62
Multi-unit structures						
Rank, percentile	0.81	0.49	0.26	0.63	0.50	0.39
Raw, %	7.5	2.9	1.4	9.4	4.7	2.1
Mobile homes						
Rank, percentile	0.22	0.50	0.79	0.00	0.51	0.75
Raw, %	4.8	10.9	20.3	0.0	0.8	7.6
Crowding						
Rank, percentile	0.48	0.45	0.52	0.45	0.50	0.53
Raw, %	1.9	1.8	2.0	1.6	1.9	2.1
No vehicle						
Rank, percentile	0.34	0.48	0.68	0.33	0.48	0.66
Raw, %	4.8	5.6	7.0	3.3	5.0	8.3
Group quarters						
Rank, percentile	0.52	0.50	0.41	0.44	0.50	0.55
Raw, %	2.1	2.0	1.7	0.2	0.3	0.4
**Overall SVI**	0.27	0.47	0.72	0.25	0.49	0.73

Abbreviation: COPD, chronic obstructive pulmonary disease; SVI, social vulnerability index.

a The 2018 CDC/ATSDR SVI includes data for 15 individual components grouped into 4 themes (socioeconomic, household composition and disability, minority status and language, and housing type and transportation) which are then summarized with an overall SVI. For each individual component, median raw estimates are calculated from original data values; median percentile ranks are calculated from percentile rankings (range, 0–1). For each of the 4 themes and the overall SVI, median raw estimates are not applicable given these are composites of multiple components; therefore, only medians of percentile rankings are reported in the rows corresponding to the 4 themes and the overall SVI.

b Pairwise rankings were significant (*P* < .01) in multiple testing at the county and census tract levels for all measures except the following: low versus moderate tract-level aged 17 or younger, moderate versus high county-level single-parent households, low versus moderate county-level housing type and transportation theme, low versus moderate and low versus high county-level crowding, moderate versus high tract-level crowding, and low versus moderate county-level group quarters.

c Total of 3,141 counties for 3 components (below poverty, unemployed, and income), the socioeconomic theme, and the overall SVI because of missing data.

d The tract-level count ranged between 72,140–72,323 (depending on the component) because of missing data.

**Figure 1:**
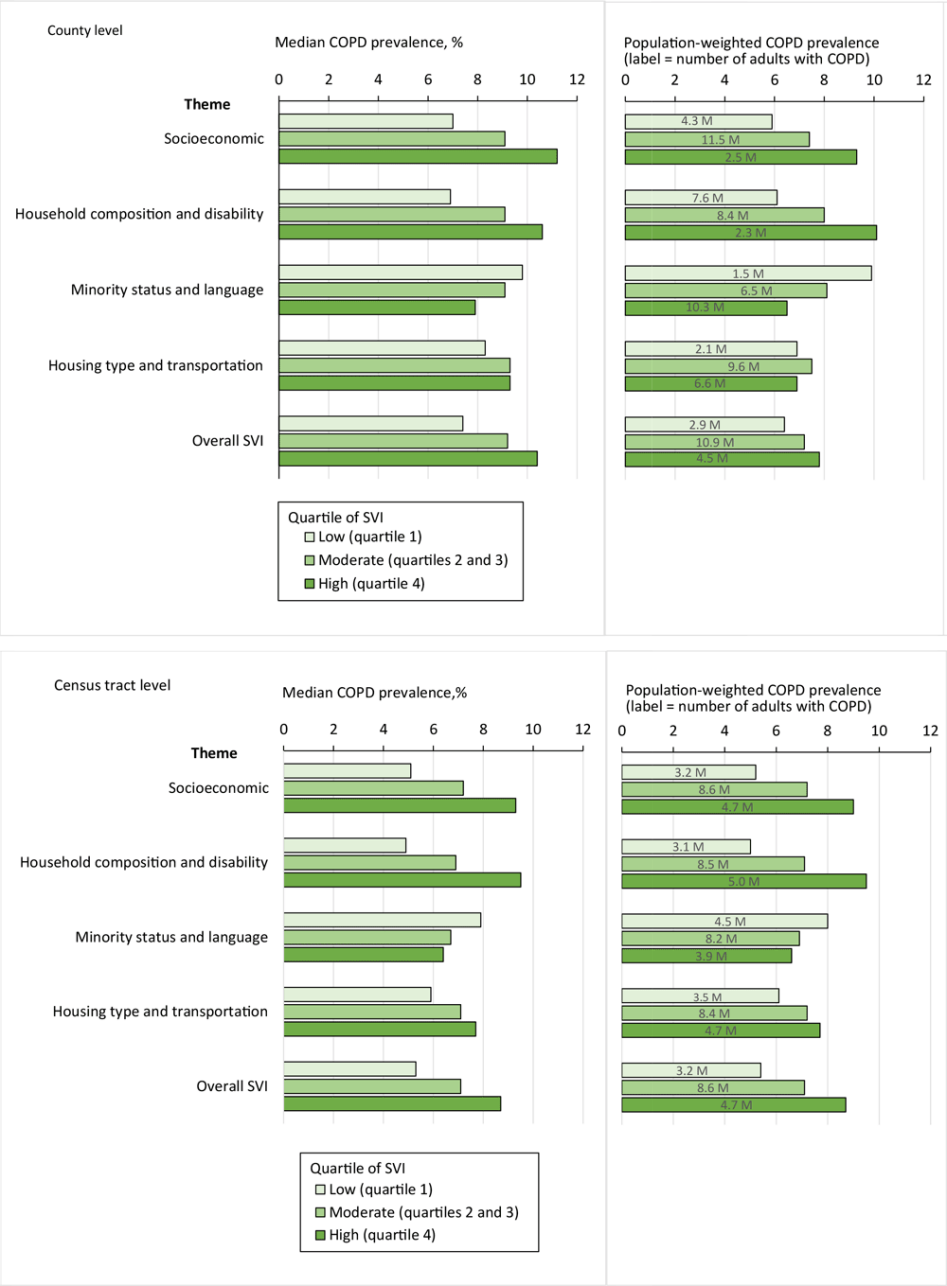
Prevalence of chronic obstructive pulmonary disease (COPD) by categories for the 4 vulnerability themes and the overall social vulnerability index (SVI) at the (a) county and (b) census tract level. Categories were based on quartiles of rankings and categorized into low (quartile 1), moderate (quartiles 2 and 3), and high (quartile 4). All pairwise differences in median county- and tract-level COPD prevalence were significantly different (*P* < .001) for the overall SVI and for all themes, except for the comparison of county-level moderate and high vulnerability for the Housing type and Transportation theme. Median and population weighted COPD prevalence estimates are shown in the figures and the estimated number of adults with COPD (in millions) are provided in the figure labels.

COPD county and tract median prevalence increased as vulnerability increased from low to high vulnerability for the socioeconomic, household composition and disability, and housing type and transportation (tract-level only) themes, and the overall SVI (Figure 1). Conversely, median prevalence decreased as vulnerability increased from low to high vulnerability for the minority status and language theme. Trends in population-weighted estimates were similar to trends using median values; however, because these were aggregate estimates, they were not statistically tested. By 2018 county-level estimates, about 18.3 million adults had COPD and 4.5 million resided in counties with high overall SVIs; by theme this ranged from 2.3 million (household composition and disability) to 10.3 million (minority status and language). By 2010 tract-level population counts, about 16.5 million adults had COPD and 4.7 million resided in tracts with high overall SVIs; by theme this ranged from 3.9 million (minority status and language) to 5.0 million (household composition and disability).

**Table:** 

Level and theme or overall index	Median COPD prevalence,%			Population weighted COPD prevalence, %			Estimated number of adults with COPD (millions)		
	Low	Moderate	High	Low	Moderate	High	Low	Moderate	High
**A. County**									
Socioeconomic theme	7.0	9.1	11.2	5.9	7.4	9.3	4.3	11.5	2.5
Household composition and disability theme	6.9	9.1	10.6	6.1	8.0	10.1	7.6	8.4	2.3
Minority status and language theme	9.8	9.1	7.9	9.9	8.1	6.5	1.5	6.5	10.3
Housing type and transportation theme	8.3	9.3	9.3	6.9	7.5	6.9	2.1	9.6	6.6
Overall SVI	7.4	9.2	10.4	6.4	7.2	7.8	2.9	10.9	4.5
**B. Census tract**									
Socioeconomic theme	5.1	7.2	9.3	5.2	7.2	9.0	3.2	8.6	4.7
Household composition and disability theme	4.9	6.9	9.5	5.0	7.1	9.5	3.1	8.5	5.0
Minority status and language theme	7.9	6.7	6.4	8.0	6.9	6.6	4.5	8.2	3.9
Housing type and transportation theme	5.9	7.1	7.7	6.1	7.2	7.7	3.5	8.4	4.7
Overall SVI	5.3	7.1	8.7	5.4	7.1	8.7	3.2	8.6	4.7

**Figure 2:**
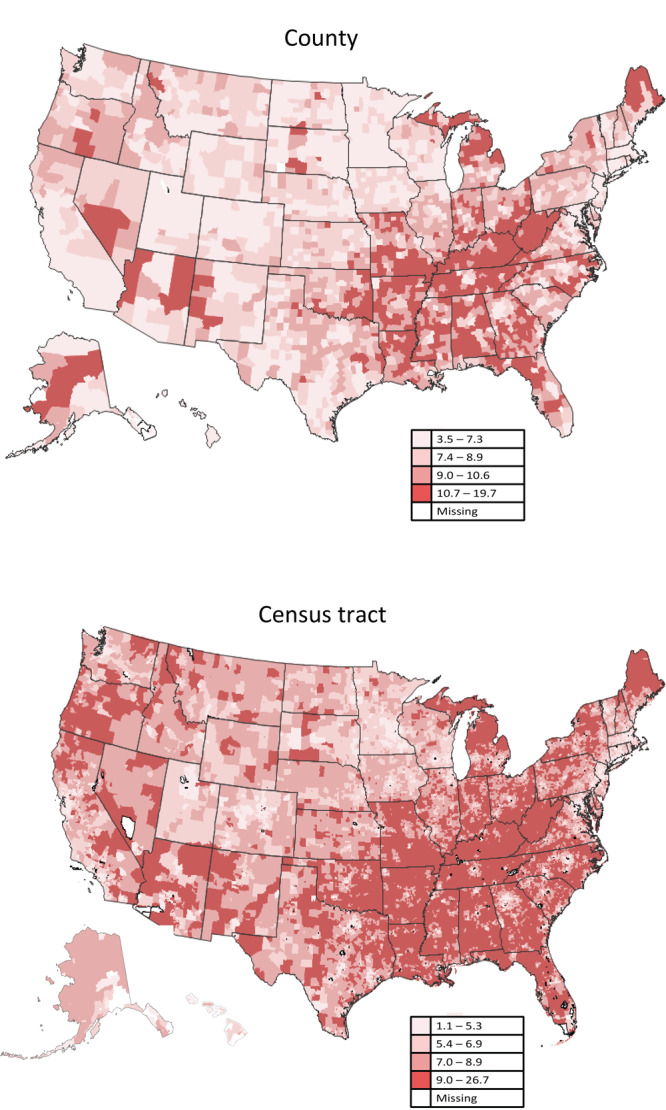
County- and census tract–level model-based prevalence estimates of chronic obstructive pulmonary disease, US, 2018. Maps were classified into 4 classes using quartiles (county: quartile 1 [3.5%–7.3%], quartile 2 [7.4%–8.9%], quartile 3 [9.0%–10.6%], quartile 4 [10.7%–19.7%]; census tract: quartile 1 [1.1%–5.3%], quartile 2 [5.4%–6.9%], quartile 3 [7.0%–8.9%], quartile 4 [9.0%–26.7%]). Source: PLACES: Local Data for Better Health, County Data 2022 release (www.cdc.gov/places).

**Figure 3:**
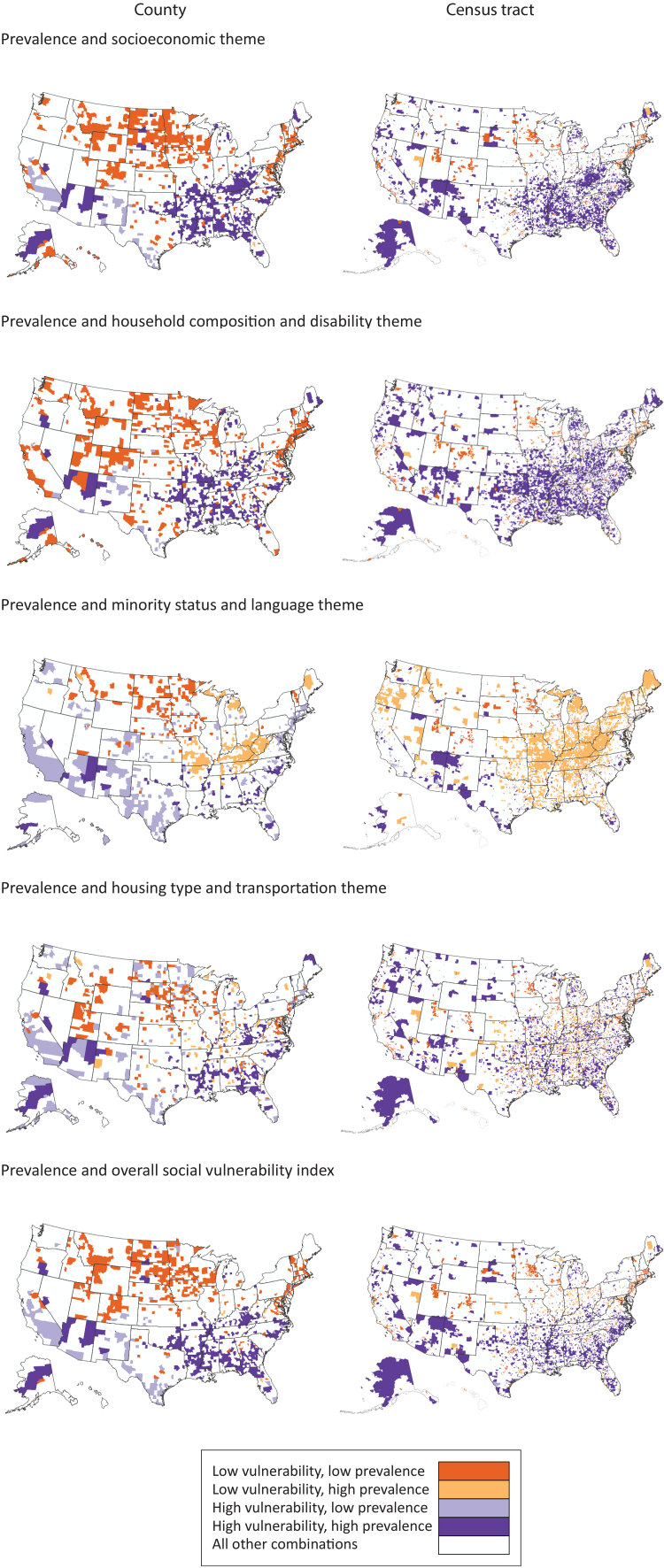
Areas of concordance (low vulnerability and low prevalence; high vulnerability and high prevalence) and discordance (low vulnerability and high prevalence; high vulnerability and low prevalence) between categories of chronic obstructive pulmonary disease prevalence and social vulnerability, US, 2018. Prevalence estimates and rankings were each categorized as low when in quartile 1 and high when in quartile 4. Concordance and discordance are mapped by county and census tract by 4 vulnerability themes: socioeconomic, household composition and disability, minority status and language, and housing type and transportation, and by the overall social vulnerability index. Source: PLACES: Local Data for Better Health (2020 data release), www.cdc.gov/places. 2018 CDC/ATSDR SVI (2020 data release) (https://www.atsdr.cdc.gov/placeandhealth/svi).

Counties and census tracts with the highest model-based COPD prevalence estimates (quartile 4) were clustered along the Ohio and lower Mississippi rivers (Figure 2). Bivariate maps showing 9 joint categories of COPD prevalence and vulnerability for the 4 themes and the overall SVI are provided in the Appendix. High COPD prevalence clustered along the Ohio and lower Mississippi Rivers at the county and tract level with high vulnerability for the socioeconomic and household composition and disability themes and the overall SVI (Figure 3). Low COPD prevalence clustered with low vulnerability for the socioeconomic theme and the overall SVI in counties in the West North Central region. There was some spatial discordance of low COPD prevalence with high vulnerability for the socioeconomic theme, housing type and transportation theme, and the overall SVI in counties in the southwest, southern California, and southern Atlantic seaboard regions. Clusters of discordance, especially clusters of high prevalence with low vulnerability, were most frequently observed for the minority status and language vulnerability theme.

## Discussion

Although tools exist for understanding data related to geographic differences in health outcomes and social vulnerability, few examples show how to combine data across sources to improve their usefulness for public health planning. By using data from PLACES and the CDC/ATSDR SVI, we demonstrated how these 2 tools can be combined to examine local-level estimates of COPD burden in the context of local-level social vulnerability. Continued efforts are needed to explore the many opportunities to combine data across tools to best support national, state, and local public health planning efforts.

By jointly examining community social vulnerability and health data, local planners and public health professionals can enhance their planning of strategies for addressing chronic conditions, including COPD. A previous study demonstrated the use of small area estimates to predict the number of adults with COPD living in the forecasted path of a hurricane (18). This demonstration study could be enhanced by adding vulnerability data that can help local areas prioritize the needs of adults with COPD within the context of a community’s broader vulnerability needs. People with COPD are medically vulnerable and ensuring their social and medical needs are met (eg, accessibility to specialists and services such as oxygen and pulmonary rehabilitation therapy) is important at both emergent and nonemergent times. This is further supported by recent literature that suggests community-level factors (eg, socioeconomic disadvantage) are associated with worse COPD-related outcomes, independent of individual-level factors (19). Our correlation findings at the geographic levels were expected because previous studies have consistently shown individual-level associations between COPD and measures of lower income, non-Hispanic White race or ethnicity, disability, and lower education levels (20–23). However, our analyses improve understanding of COPD and social vulnerability from a public health standpoint by demonstrating the connectivity of these factors within a community. This understanding can be an important part of needs assessments and can enhance planning and implementation of strategies to address COPD and social vulnerability.

We identified areas of concordance and discordance between high and low COPD prevalence and vulnerability. Identifying these types of clusters can help guide public health planning; however, other factors may contribute to the clustering. First, the PLACES statistical approach incorporates sex, age, race and ethnicity, education, and county-level poverty when generating estimates (5,14). It is important for users to understand the modeling process so they can better describe how an area’s demographic characteristics influence estimates and may contribute to the geographic disparities observed across areas. Also, the modeling approach could generate estimates that potentially over- or underestimate COPD’s association with these variables. Although research has found that model-based small area estimates and direct survey estimates of COPD prevalence are consistent at the county and state level (14), less is known about consistency at the census tract level or if consistency based on the characteristics of a geographic area may differ. If reliable and directly measured local-level data on COPD burden or more contextual SDOH measures are available, these data can also be incorporated into planning efforts, as can data on the availability of health care providers (eg, pulmonologists, primary care physicians) (24) and treatment facilities (eg, pulmonary rehabilitation facilities). Second, in our study, we focused on bivariate analysis, but other components may contribute to concordance and discordance. For example, when scanning PLACES estimates of smoking, a major risk factor for COPD, many areas we identified with clusters of discordance had lower estimates of current smoking. However, our study did not examine these other interrelated components. The CDC/ATSDR SVI and PLACES are valuable tools to guide public health planning, especially where direct local data are unavailable; however, when using these data in the planning and decision-making process, it is important to understand potential limitations and ensure steps are taken to consider population characteristics, local context, and additional interrelated factors as part of the process.

We presented a case study at the national level to demonstrate how these 2 data sets can be combined to inform public health planning. However, efforts to replicate these analyses within more focused geographies (eg, counties or tracts within a state, tracts within a county) could help better inform state and local efforts in planning and implementing strategies for COPD prevention (eg, smoking cessation), early diagnosis, treatment (eg, pulmonary rehabilitation, oxygen therapy, medications), and management (eg, approaches to slow declining lung function, prevent exacerbations). Our overall findings were similar regardless of whether county or tract data were examined, with one exception: estimates of the total number of adults with COPD living in areas defined as having low, moderate, or high vulnerability differed when using county versus tract estimates. The reason for this is likely the wider range in county population (2020 population estimates for adults ranged from 88 to 7,916,625; median, 20,212 [3,142 counties]) compared with the range in tract population (2010 tract population counts for adults ranged from 16 to 29,214; median, 3,067 [72,337 tracts included in PLACES]). For example, counties with large populations could be highly influential when calculating aggregate population estimates. For generating aggregate population estimates, it may be best to use the smallest geographic unit for which the measures are available.

All data used in these analyses were downloaded from public data portals. Other options for jointly examining PLACES and CDC/ATSDR SVI data include opening both data sets or maps and looking at them side by side or using available services in the ArcGIS Living Atlas of the World (https://livingatlas.arcgis.com). Several interactive tools also provide access to measures of health outcomes along with social and economic factors within a single application or include functions that allow users to stratify maps of health outcomes by ranges of SDOH measures or vice versa (eg, CDC’s Interactive Atlas of Heart Disease and Stroke [8], County Health Rankings & Roadmaps [25], US Diabetes Surveillance System [9]).These different options offer starting points for incorporating both health and SDOH measures in public health planning tools. Understanding more about the types of analysis and visualizations needed by local public health planners can help inform the development of the next generation of interactive tools to jointly examine measures of SDOH and chronic disease.

Similar analyses could be conducted to inform efforts that focus on other health outcomes (eg, arthritis, depression), health risk behaviors (eg, binge drinking, sleep), prevention practices (eg, annual check-ups, screenings), or health status (eg, physical or mental health) that are included as PLACES measures. As analyses plans are developed, it is important to consider existing knowledge related to the health measure and consider how to conceptualize a joint analysis to best inform the planning and decision-making process (26). In addition, measures of social vulnerability or social determinants of health from other sources could be incorporated (eg, AHRQ’s Social Determinants of Health Database [available at county and ZCTA level], US Census Bureau Community Resilience Estimates [available at county and tract level]) (10). CDC provides several compilations of data sets, and tools and resources to help practitioners address SDOH (7,10).

We provided an example of using estimates from 2 validated CDC data sources for public health planning; however, our approach has several limitations. First, both tools rely on estimates based on samples of the population. All estimates have associated margins of error, and estimates can be affected by biases from the surveys themselves (eg, recall, sampling). We did not include measures of error in our analysis and did not provide CIs for aggregate estimates. Second, the PLACES approach has limitations, which are discussed elsewhere (5,14), and the modeling approach could affect the correlations observed, as discussed previously in this article. In addition, the census tract COPD prevalence estimates used in this study were generated by using 2010 census population counts, which creates a time disconnect between the PLACES and CDC/ATSDR SVI estimates. Third, our study used an ecologic design to identify and describe how 2 factors of interest (COPD prevalence and social vulnerability) co-exist across geographies. This design is susceptible to the “ecological fallacy” and is not appropriate for examining independent or causal effects; nor does it allow separate examination of adults with COPD. Fourth, the CDC/ATSDR SVI is limited to social factors. Although it compared well with other indexes as a guide to disaster preparation (27), these factors are only one component of understanding a community’s vulnerability. For example, adults with COPD are also medically vulnerable because they are more likely to need access to special equipment (eg, portable oxygen tanks), have more comorbid chronic conditions requiring care, and have more hospitalizations and emergency department visits because of exacerbations (22,28,29). Adding local measures of medical vulnerability could create a more complete picture of a community’s vulnerability and better inform community planning activities.

We demonstrated how data from 2 publicly available tools can be combined, analyzed, and visualized to jointly examine local COPD estimates and social vulnerability. By understanding this approach and improving awareness of these tools, public health practitioners can enhance their use of these data to inform public health planning to better inform actions to improve health.

## Appendix Supplemental Figure

Bivariate maps show county- and census–tract-level categories of chronic obstructive pulmonary disease (COPD) prevalence with social vulnerability in the US in 2018. County and census tract bivariate maps are displayed for each of the 4 vulnerability themes: socioeconomic, household composition and disability, minority status and language, and housing type and transportation, and by the overall social vulnerability index (SVI). Prevalence estimates and vulnerability rankings were each categorized into low (quartile 1), moderate (quartiles 2 and 3), and high (quartile 4). Areas are presented cross-classified into 9 categories representing 3 categories of COPD prevalence by 3 categories for each theme and the overall SVI. (Sources: PLACES: Local Data for Better Health [2020 data release], www.cdc.gov/places; 2018 CDC/ATSDR SVI (2020 data release), https://www.atsdr.cdc.gov/placeandhealth/svi.)

**Text version of supplemental figure:** Five US county maps and 5 US census tract maps display 3 × 3 bivariate maps of 3 levels of COPD prevalence (low, moderate, and high) by 3 levels (low, moderate, and high) of each of the 4 vulnerability themes (socioeconomic, household composition and disability, minority status and language, housing type and transportation) and the overall social vulnerability index. The map shows clusters of counties and tracts along the Ohio and lower Mississippi rivers in the high COPD prevalence and high vulnerability category for the socioeconomic, household composition and disability, and overall index.
